# Oral health-related quality of life of paediatric patients with AIDS

**DOI:** 10.1186/1472-6831-11-2

**Published:** 2011-01-05

**Authors:** Débora B Massarente, Carina Domaneschi, Heloísa HS Marques, Samantha B Andrade, Daniela Goursand, José LF Antunes

**Affiliations:** 1 Faculdade de Odontologia da Universidade de São Paulo, 2227 Av Prof Lineu Prestes, 05508-900, Sao Paulo, Brazil; 2 Instituto da Criança, Faculdade de Medicina da Universidade de São Paulo, 647 Av Dr Enéas Carvalho de Aguiar, 05403-000, Sao Paulo, Brazil; 3 Faculdade de Odontologia da Universidade Federal de Minas Gerais, 6627 Av Antonio Carlos, 31270-901, Belo Horizonte, Brazil; 4 Faculdade de Saúde Pública da Universidade de São Paulo, 715 Av Dr Arnaldo, 01246-904, Sao Paulo, Brazil

## Abstract

**Background:**

Children with Acquired Immune Deficiency Syndrome (AIDS) exhibit impaired dental status, which can affect their quality of life. This study assessed the oral health-related quality of life of these patients and associated factors.

**Methods:**

The "Child Perceptions Questionnaire 11-14", rating overall and domain-specific (oral symptoms, functional limitations, emotional well being, and social well being) oral health-related quality of life (OHR-QoL) was completed by 88 children with AIDS assisted in the Child Institute, Sao Paulo, Brazil. Parents or guardians provided behavioural and socio-demographic information. The clinical status was provided by hospital records. OHR-QoL covariates were assessed by Poisson regression analysis.

**Results:**

The most affected OHR-QoL subscale concerned oral symptoms, whose rate was 23.9%. The direct answer for oral health and well being made up a rate of 47.7%. Brushing the teeth less than two times a day and viral load exceeding 10,000 HIV-RNA copies per millilitre of plasma were directly associated (p < 0.05) with a poorer oral health-related quality of life.

**Conclusions:**

Children with more severe AIDS manifestations complained of poorer status of oral symptoms, functional limitations, emotional and social well being related to their oral health. Recognizing the factors that are associated with poorer OHR-QoL in children with AIDS may contribute to the planning of dental services for this population.

## Background

In spite of being an avoidable condition, the perinatal, vertical transmission of human immunodeficiency virus (HIV) affected from 25 to 50% of children born to infected mothers during the 1980s in Brazil [1]. The first case in a Brazilian child was reported in 1983. By 2006, the number of HIV-infected children who were less than 13 years old and manifested AIDS symptoms totalled 19,825 [2].

More than 10% of paediatric patients who have AIDS in Brazil live in São Paulo [2]. São Paulo is the largest and most industrialized city in the country, and is located at the heart of an even larger metropolitan area. Therefore, its healthcare system is required to assist dwellers of neighbouring towns, and even inhabitants of other cities within the State of São Paulo, and other Brazilian states, because the city contains nationwide reference units for several healthcare specialties. HIV-infected children and those who already manifest AIDS symptoms need continuous medical care and monitoring of health status. In particular, these children may need special care dentistry for oral health promotion.

Poor socioeconomic status, dental caries, reduced immunological response, painful symptoms in the mouth, decreased salivary function and continuous use of medicines that have been formulated as syrups or sugared solutions are usual characteristics of children with AIDS [3,4]. These children frequently exhibit lesions on the oral mucosa, resulting in pain that contributes to ineffective or infrequent tooth brushing. All of these conditions imply the risk of a prejudicial impact on oral health, and may affect the quality of life (QoL).

Notwithstanding the foregoing, the access of paediatric patients who have AIDS or are HIV positive to programs of oral health is made difficult by the stress of parents and guardians, the accumulation of multiple treatment needs, and reduced integration of units of medical and dental care. The insufficient provision of dental services for children with special care needs has been appraised as a chronic public health problem [5].

This study sought to gather relevant information to plan oral health programs that target children with AIDS. The primary objective of the study was to assess the self perception of oral health by HIV positive paediatric patients who already manifest AIDS symptoms. The secondary objective was to assess the associated socio-demographic, behavioural and clinical factors.

## Methods

A total of 88 children who were 10 to 15 years old participated in this study. All of these children were enrolled for continued medical monitoring at the outpatient unit of the Child Institute of the School of Medicine, University of Sao Paulo, Brazil, and met the criteria for AIDS case definition - an HIV positive diagnosis, and manifestation of AIDS symptoms with moderate or severe immunologic suppression (low CD4+ cell count for age) [6]. All children who participated in this study were affected by HIV vertical transmission. Exclusion criteria comprised denial to participate and failing to attend hospital scheduled consultations in the period of data gathering.

The study protocol observed international statutes and national legislation on ethics in research that involves human beings. All patients consented to participate in the study and their parents or guardians signed a term of consent. The study protocol was approved by the participating institutions' committees (School of Medicine and School of Dentistry, University of Sao Paulo).

From March to August, 2009, these children completed the Child Perceptions Questionnaire for 11-14-year-old children - CPQ11-14 [7], a form that was specifically designed to assess oral health-related quality of life (OHR-QoL) in this age group. It had already been translated to Portuguese and validated for use in Brazil [8]. Although the questionnaire was originally proposed for 11-14-year-old children, this study included 10- and 15-year-old children with the intention of recruiting more participants, and producing more information. The children completed the questionnaires by themselves. However, the interviewer remained nearby, to be available in case there were any uncertainties. If any questions remained unanswered, the interviewer readdressed the question, explaining its meaning and annotating the answer.

The questionnaire contained two overall indices on oral health and wellbeing ("Would you say that the health of your teeth, lips, jaws and mouth is...?" and "How much does the condition of your teeth, lips, jaws or mouth affect your life overall?"), and 37 specific questions on the following domains of OHR-QoL: oral symptoms, functional limitations, emotional wellbeing, and social wellbeing. Each question was answered by selecting one of five alternative responses. These alternatives were assigned values of 0 to 4, with the higher values corresponding to a poorer QoL [7,8]. For example, the categories for the first question ranged from very poor (score = 4) to excellent (score = 0). The rates for each domain were standardized from 0 to 100%, thus allowing comparisons between subscales that differed in the numbers of questions. This comparison used the non-parametric test of Kruskal-Wallis. The overall scale (37 questions) and the two overall indices on oral health and well being were not compared to the subscale rates, due to the large difference in the number of questions.

Information related to clinical status was obtained from medical records kept on file by the hospital. International standards that have been adopted in Brazil determined the classification of symptom severity in paediatric patients with AIDS [6]. Information on viral load and CD4+ T-lymphocytes were also gathered. Viral load is quantified by the number of HIV-RNA copies per millilitre of plasma. The cut-off point for categorical classification (10,000 copies) is high, and delimitates an advanced stage of the infection. The classification of CD4+ cell count differentiated those children who presented with a low number for their ages: less than 500 cells for children from 10 to 12 years old, and less than 350 for patients aged 13 to 15 years old. Values lower than these cut-off points are considered to be indicative of moderate or severe immunologic suppression.

Parents and guardians answered a supplementary questionnaire on socioeconomic and behavioural variables. Families were classified according to characteristics of their households - ownership (yes or no) and crowding, as assessed by the ratio of inhabitants per room, dichotomously classified (>1.0; ≤1.0). The caregiver of the family (mother or other relatives) and toothbrush frequency (twice per day or more frequently, less than twice per day) were the behavioural variables that were selected for this study.

The results were tabulated and statistically analyzed using the Stata 10.0 software (Stata Corporation, College Station, TX, USA). The association between OHR-QoL rates and socioeconomic, behavioural and clinical factors was assessed by Poisson regression analysis, thus estimating rate ratios among comparison groups and their respective 95% confidence interval. All regression models were adjusted for age, because individual answers to the questionnaire were previously reported to be dependent on age [9], despite the reduced variation in age within the group of participants.

## Results

Ninety seven (10 to 15 years old) patients, who were enrolled in the healthcare unit, were eligible for this study. Two children refused to participate, and seven were considered to have dropped out because they had not attended the hospital scheduled consultations. The remaining 88 children (43 girls and 45 boys) participated in the study. For most of them (57%), their mother was the family caregiver; 41% of them manifested severe symptoms of AIDS (Table 1).

**Table 1:** Socio-demographic, behavioural and clinical conditions of 88 children with AIDS, São Paulo, 2009.

Children with AIDS, São Paulo, 2009		N (%)
***Socio-demographic variables***		

Gender	Girls	43 (49)
	Boys	45 (51)
Household crowding (1)	Yes	24 (27)
	No	64 (73)
Household ownership	Yes	56 (64)
	No	32 (36)

*Health behaviour*		

Who is the caretaker	Others	38 (43)
	Mother	50 (57)
Toothbrush	2 or more times daily	73 (83)
	less than 2 times daily	15 (17)

*AIDS-related variables*		

Clinical status	severe symptoms	36 (41)
	absent, mild or moderate symptoms	52 (59)
CD4+ count (2)	low for age	36 (41)
	normal for age	52 (59)
Viral load (3)	≥10,000	25 (28)
	< 10,000	63 (72)

(1) Ratio (inhabitants per rooms) higher than one.

(2) CD4+ T-lymphocyte cells per cubic millimetre of blood.

(3) HIV-RNA copies per millilitre of plasma.

Table 2 shows average and median rates for two overall indices of oral health and well being, for the total scale and each subscale in the questionnaire. Standardized rates allowed oral symptoms to be identified as the most affected domain of OHR-QoL, with rates scoring significantly higher (p < 0.05) than in the remaining subscales. When the two overall indices on oral health and well being were compared, the first ranked significantly higher (p < 0.05) than the second.

**Table 2:** Overall indices on oral health and well being; total and subscale CPQ11-14 rates of 88 children with AIDS, São Paulo, 2009.

CPQ11-14	Range of possible rates (1)	Range of observed rates	Median rates	Mean rates (SD)	Standardized rates (2)
Index 1. "Would you say that the health of your teeth, lips, jaws and mouth is...?"	0-4	0-4	2.0	1.9 (1.1)	47.7%
Index 2. "How much does the condition of your teeth, lips, jaws or mouth affect your life overall?"	0-4	0-4	1.0	1.4 (1.3)	34.1%
Total scale (37 items)	0-148	2-90	25.5	28.5 (17.9)	19.2%

Subscales					
Oral symptoms (6 items)	0-24	0-18	5.0	5.7 (3.4)	23.9%
Functional limitations (10 items)	0-40	0-20	7.0	7.8 (4.7)	19.4%
Emotional well-being (9 items)	0-36	0-30	4.5	7.0 (6.9)	19.4%
Social well-being (12 items)	0-48	0-33	6.0	8.0 (7.1)	16.7%

(1) Each rate ranges from 0 (best subjective status) to 4 (worst self-reported condition).

(2) Rates were standardized to range from 0 (best subjective status) to 100% (worst self-reported condition).

Table 3 synthesizes the study of factors that are associated with the overall indices of oral health and well being. Girls reported a higher impact in the first question than boys. The same was observed for children who lived in more crowded households and for children who did not brush their teeth at least twice per day. No covariate assessed in this study were significantly associated with answers to the second question. The assessment of the overall scale showed a higher impact on children with a lower frequency of tooth brushing, and on those with a poorer clinical status (as assessed by a higher HIV viral load).

**Table 3:** Assessment of factors associated with overall indices on oral health and well being and total CPQ11-14 rates of 88 children with AIDS, São Paulo, 2009.

CPQ11-14		Question 1	RR(95%CI) (1)	Question 2	RR(95%CI) (1)	Total scale	RR(95%CI) (1)
		Mean (2)	significance	Mean (3)	significance	Mean	significance
*Socio-demographic characteristics*		Mean (2)	significance	Mean (3)	significance	Mean	significance

Gender	Girls	2.2	1.30 (1.01-1.67)	1.3	0.88 (0.60-1.30)	29.2	1.03 (0.79-1.33)
	Boys	1.7	P = 0.040*	1.4	P = 0.520	27.8	P = 0.833
Household crowding (2)	Yes	2.3	1.30 (1.01-1.67)	1.3	0.99 (0.65-1.50)	29.1	1.04 (0.76-1.42)
	No	1.8	P = 0.041*	1.4	P = 0.967	28.3	P = 0.792
Household ownership	Yes	2.0	1.09 (0.82-1.44)	1.3	0.87 (0.58-1.31)	26.7	0.93 (0.71-1.22)
	No	1.8	P = 0.566	1.5	P = 0.509	29.9	P = 0.620

*Health behaviour*							

Who is the caretaker	Other	2.1	1.17 (0.92-1.48)	1.6	1.27 (0.87-1.87)	31.4	1.20 (0.93-1.55)
	Mother	1.8	P = 0.200	1.2	P = 0.215	26.3	P = 0.171
Toothbrush	2 or more	1.5	0.71 (0.55-0.91)	1.4	1.35 (0.78-2.35)	26.6	0.68 (0.50-0.91)
	Less than 2	2.2	P = 0.007*	1.0	P = 0.279	37.3	P = 0.009*

*AIDS-related variables*							

Clinical status	Severe symptoms	2.1	1.19 (0.94-1.52)	1.3	0.88 (0.60-1.29)	31.0	1.15 (0.89-1.48)
	Absent, mild or moderate symptoms	1.8	P = 0.155	1.4	P = 0.507	26.7	P = 0.300
CD4+ count (3)	Low for age	2.1	1.17 (0.92-1.48)	1.4	1.00 (0.68-1.49)	29.6	1.02 (0.79-1.31)
	Normal for age	1.8	P = 0.194	1.3	P = 0.986	27.7	P = 0.897
Viral load (4)	≥10,000	2.1	1.13 (0.88-1.46)	1.3	0.94 (0.59-1.48)	37.8	1.54 (1.20-1.99)
	< 10,000	1.8	P = 0.338	1.4	P = 0.776	24.8	P = 0.001*

* p < 0.05.

(1) Rate ratio (95% confidence interval), adjusted for age.

(2) "Would you say that the health of your teeth, lips, jaws and mouth is...?"

(3) "How much does the condition of your teeth, lips, jaws or mouth affect your life overall?"

(4) Inhabitants per room ratio higher than one.

(5) CD4+ T-lymphocyte cells per cubic millimetre of blood.

(6) HIV-RNA copies per millilitre of plasma.

Higher HIV viral load also associated with poorer ranking in all subscales (Table 4). Having a low CD4+ count (in comparison to reference values for the corresponding age) associated with poorer answers for questions that assessed functional limitations. An improved socioeconomic condition - household ownership - associated with better rates in the scale that assessed oral symptoms. Children that could not count on their own mother as a caregiver scored lower on the social well being subscale. A lower frequency of tooth brushing was significantly associated with worse answers in the functional limitations and emotional well being subscales.

**Table 4:** Assessment of factors associated with subscale CPQ11-14 rates of 88 children with AIDS, São Paulo, 2009.

CPQ11-14		Oral symptoms	RR(95%CI)(1)	Functional limitations	RR(95%CI) (1)	Emotional wellbeing	RR(95%CI) (1)	Social wellbeing	RR(95%CI) (1)
		Mean	significance	Mean	significance	Mean	significance	Mean	significance
*Socio-demographic characteristics*									

Gender	Girls	5.3	0.85 (0.67-1.08)	8.1	1.08 (0.83-1.39)	7.3	1.07 (0.71-1.60)	8.5	1.09 (0.76-1.57)
	Boys	6.1	P = 0.186	7.4	P = 0.571	6.7	P = 0.760	7.6	P = 0.642
Household crowding (2)	Yes	6.0	1.08 (0.81-1.44)	8.2	1.08 (0.82-1.41)	7.3	1.09 (0.65-1.78)	7.8	0.95 (0.62-1.46)
	No	5.6	P = 0.600	7.6	P = 0.599	6.8	P = 0.736	8.2	P = 0.803
Household ownership	Yes	5.2	0.78 (0.60-1.00)	7.4	0.89 (0.68-1.15)	7.1	1.07 (0.69-1.64)	8.0	1.00 (0.69-1.45)
	No	6.7	P = 0.050*	8.4	P = 0.370	6.8	P = 0.772	8.1	P = 0.982

*Health behaviour*									

Who is the caretaker	Other	5.9	1.07 (0.83-1.37)	8.5	1.18 (0.91-1.52)	7.0	1.00 (0.66-1.51)	10.0	1.54 (1.08-2.18)
	Mother	5.6	P = 0.593	7.2	P = 0.209	7.0	P = 0.999	6.5	P = 0.017
Toothbrush	2 or more	5.4	0.74 (0.53-1.03)	7.4	0.74 (0.57-0.97)	6.1	0.51 (0.32-0.80)	7.7	0.75 (0.52-1.08)
	Less than 2	7.1	P = 0.076	9.7	P = 0.031*	11.3	P = 0.003*	9.7	P = 0.121

*AIDS-related variables*									

Clinical status	Severe symptoms	5.8	0.99 (0.78-1.26)	8.3	1.13 (0.87-1.46)	7.6	1.13 (0.75-1.71)	9.4	1.30 (0.90-1.87)
	Absent, mild or moderate symptoms	5.7	P = 0.964	7.3	P = 0.363	6.6	P = 0.558	7.1	P = 0.160
CD4+ count (3)	Low for age	5.2	0.80 (0.62-1.03)	9.4	1.41 (1.12-1.78)	7.1	0.96 (0.63-1.45)	7.9	0.92 (0.90-1.87)
	Normal for age	6.1	P = 0.080	6.6	P = 0.004*	6.9	P = 0.837	8.1	P = 0.658
Viral load (4)	≥10,000	7.1	1.39 (1.07-1.82)	10.0	1.46 (1.14-1.87)	9.8	1.69 (1.12-2.54)	10.9	1.61 (1.11-2.34)
	< 10,000	5.2	P = 0.015*	6.9	P = 0.003*	5.9	P = 0.013*	6.9	P = 0.012*

* p < 0.05

(1) Rate ratio (95% confidence interval), adjusted for age.

(2) Inhabitants per room ratio higher than one.

(3) CD4+ T-lymphocyte cells per cubic millimetre of blood.

(4) HIV-RNA copies per millilitre of plasma.

## Discussion

HIV positive children have been reported to suffer a higher prevalence of dental caries [3,4], candidiasis, leukoplakia, herpetic lesions, lymphadenopathy and parotiditis than other children [10,11]. The clinical status of children participating in this study, as obtained from the dental examination, was reported in another study [4]. However, little is known about the functional, emotional and social consequences of poor oral health among these children [12]. Having assessed the self-perception of paediatric patients with AIDS, this study reported socio-demographic, behavioural and clinical factors that are associated with a higher impact on OHR-QoL. These are the main results of this study, and they may instruct programmes that are undertaken to monitor the oral health of children who have AIDS.

General questions about self-perceived health are a useful resource that is commonly used in surveys. Endorsed by the World Health Organization [13], this strategy permits the production of health indices that are related to several variables and contribute to an assessment of the demand and effective use of healthcare services. This proposition highlights the importance of including overall indices of oral health and well being in the questionnaire used in this study. Oddly, discrepant results were obtained by questions 1 and 2. Question 1 (on the overall perception of oral health) revealed a higher prejudicial impact than question 2 (on how much oral health affects life overall). Furthermore, answers to question 1 were related to socio-demographic and behavioural factors, whereas answers to question 2 did not.

The comparative analysis of subscale rates showed oral symptoms as the QoL domain that was most affected in children examined in this study. This conclusion is consistent with previous studies that report the prejudicial impact of oral lesions on the OHR-QoL of adults who were affected [14] and unaffected [15] by the HIV infection. Fostering the importance of this observation, we observe that dental caries, soft tissue lesions and infections in the oral cavity are acknowledged to be frequent manifestations in paediatric patients with AIDS [3,4,10-12].

Methodological tools that systematize how children assess their own oral health are relatively recent and, as yet, no reference averages for CPQ11-14 rates have been established for the overall population. Indeed, conflicting conclusions were observed from the comparison of our data with those of previous studies that assessed OHR-QoL in Brazilian children. Currently assessed averages for subscale and overall rates ranked higher than those reported for 114 children without AIDS and free of caries, and live in another Brazilian city (Belo Horizonte) [8]. However, the results for 55 children enrolled in public schools in Piracicaba (State of São Paulo) [9], who have not AIDS and were free of caries, were similar to those reported in this study.

In regards to the identification of associated factors, it is noteworthy that tooth brush frequency was virtually indicative of protection against prejudicial impacts on OHR-QoL. This observation is in agreement with the hypothesis that a simple, although effective resource of oral health promotion can contribute to improve QoL. However, as this study exclusively assessed cross-sectional information, one cannot rule out a reverse causality, hypothesizing that children with AIDS who feel a higher impact of poor oral health may have greater difficulty to maintain a desirable tooth brushing frequency.

This study also highlights the importance, for these children, of having their own mothers as caregivers. This also reinforces the importance of home monitoring as an effective resource in oral health promotion, which is in agreement with the observation of household crowding as an additional factor that impacts on OHR-QoL. Household overcrowding has been used as a proxy for socioeconomic status in epidemiological studies assessing dental disease [16], because poorer subjects in Brazil tend to live in more crowded households.

Viral load was the most relevant clinical indicator in the assessment of OHR-QoL. This is relevant for healthcare units that attend children with AIDS, and must be taken into consideration in the planning of both medical and dental services for this group of patients. Children who presented an advanced stage of HIV infection (more than 10,000 HIV-RNA copies per millilitre of plasma) ranked poorer in OHR-QoL overall scale and in the four subscales. This information should be taken into consideration when planning dental services, to anticipate interventions that potentially result in QoL improvements for these patients.

The OHR-QoL questionnaire was originally designed for children of ages 11 to 14 years; but the current study included 10- and 15-year-old patients in order to recruit a higher number of participants and to improve the assessment of associated factors. Self-perception of oral health, however, has been described as dependent on age [9], and the enlargement of the age range may have modified the profile of answers. However, a regression analysis that is adjusted for age may have controlled, at least in part, the effect of age variation on the identification of factors that modify OHR-QoL.

In spite of having expanded the intended age range of respondents for the questionnaire (from 11-14 to 10-15 years old), the sample size (88 children) was still reduced, and may have had an insufficient number of participants to assess other factors that eventually associate with the study outcome. In addition, the study group corresponds to one paediatric hospital, and cannot be considered to be representative of children with AIDS in the Brazilian context. Sampling design - restricted to a single hospital and with a relatively reduced number of participants - is acknowledged as the main study limitation. Therefore, this study strongly advocates the realization of further studies on OHR-QoL, with broader and more representative samples of children with AIDS.

Participating children completed the questionnaire in a separate room of the medical setting, immediately after they had their clinical consultation. We wonder whether they would have answered differently if they have been assessed in their homes or schools, and we are unaware of evidence on this issue. Assessing these children in the medical facility that they attend was a methodological option that facilitated the access to eligible participants; although this option may entail an additional study limitation.

The assessment of OHR-QoL is an important adjunct to the establishment of priorities and planning of healthcare programs. Since the 1980 s, patients have been invited to report their perception of health in evaluations of therapeutic and prophylactic resources, and in the clinical decision making [17]. However, the specific questionnaire used in this study was only proposed in 2002 [7], and is recognized as the first survey instrument specifically designed to assess self-perceived oral health in children [18].

The perspective of assessing children's perception of oral health is even more recent in Brazil. This questionnaire was translated and validated in 2008 [8,9]. To our knowledge, the current study was the first one to assess OHR-QoL in children with AIDS in the Brazilian context [19]. Assessing the magnitude of prejudicial impacts of oral health on QoL, and reporting associated factors may relevantly contribute to the planning of appropriate programs of dental services to children who have AIDS, thus contributing to an improvement of their oral and systemic health.

## Conclusions

AIDS-related clinical characteristics associated with more severe impacts on OHR-QoL. Children with more severe AIDS manifestations complained of a poorer status of oral symptoms, functional limitations, emotional and social well being related to their oral health. Brushing the teeth two or more times a day and having their own mother as caretaker associated with improved oral health related quality of life, which reinforce the importance of the attention that these children receive in their own household. This study's findings highlight the need to integrate the dentist in the interdisciplinary health care team that assists paediatric patients with AIDS, and can instruct health programs that are intended to improve their overall quality of life.

## Competing interests

The authors declare that they have no competing interests.

## Authors' contributions

DBA, CD and JLFA designed the study, gathered the information, performed the statistical analysis and wrote the first draft of the manuscript. HHSM and SBA monitored the gathering of data and statistical analysis. DG designed the form for data gathering and supervised the statistical analysis. All authors read and approved the final manuscript.

## Pre-publication history

The pre-publication history for this paper can be accessed here:

http://www.biomedcentral.com/1472-6831/11/2/prepub
